# Investigating the association between koala retrovirus and primary bone neoplasia in koalas (*Phascolarctos cinereus)* using real-time PCR and a novel immunohistochemistry assay

**DOI:** 10.1177/03009858251403173

**Published:** 2025-12-26

**Authors:** Carmen Chu, Lee McMichael, Joanne Gordon, Chiara Palmieri, Joanne Meers, Joerg Henning, Viviana Gonzalez-Astudillo

**Affiliations:** 1 School of the Environment, The University of Queensland, St. Lucia Campus, St. Lucia, QLD, Australia; 2 School of Veterinary Science, The University of Queensland, Gatton Campus, Gatton, QLD, Australia

**Keywords:** bone, chondrosarcoma, immunohistochemistry, koala, neoplasia, osteochondroma, osteosarcoma, PCR, retrovirus, tumor

## Abstract

Koala retrovirus (KoRV) is a significant infectious agent impacting the health of wild and captive koala populations worldwide. While previous studies have explored its association with neoplastic diseases such as leukemia and lymphoma, its potential involvement in primary bone tumors remains unclear. This study aimed to expand our understanding of KoRV’s disease spectrum by examining its potential association with primary bone tumors in koalas. Koala retrovirus proviral DNA load was analyzed in neoplastic bone using real-time quantitative polymerase chain reaction (PCR) and compared to healthy bone samples across 4 different gene targets: KoRV *pol*, KoRV-A *env*, KoRV-B *env*, and KoRV-D *env*. The relative KoRV subtype A proviral load was significantly lower in bone tumor samples (*n* = 14) when compared to healthy bone samples (*n* = 11) (*P* = .025), while other subtype-specific proviral loads did not differ significantly between tumor and healthy controls. In addition, we developed a novel immunohistochemistry assay to detect the KoRV capsid protein. Immunolabeling revealed KoRV capsid protein expression in all bone tumor samples (14/14, 100%), with an overall mean positive immunolabeling of 50.4% of tumor cells. The bone tumor group had a higher median H-score compared to the control group (*P* < .001). Among tumor subtypes, the highest mean percentage of tumor cell labeling was observed in osteosarcomas (73.0%), followed by chondrosarcomas (51.6%) and osteochondromas (38.0%). Collectively, these findings suggest that KoRV may have an important role in koala bone tumor oncogenesis, warranting further investigation into its potential as a contributing factor in tumor development.

Disease is a leading cause of mortality in certain koala populations and contributes to the population decline in this species, along with habitat degradation, trauma due to motor vehicle accidents and dog attacks, and climate change.^11,12,29,35^ The most important infectious agents affecting koalas are *Chlamydia* spp. and koala retrovirus (KoRV), the only known retrovirus to be currently undergoing endogenization into a vertebrate genome.^7^

KoRV is a relatively recently discovered gammaretrovirus impacting wild and captive koala populations with significant welfare and conservation implications.^13^ Currently, there are 12 described KoRV subtypes (KoRV-A to I and K to M) divided into 3 major clades.^3,6^ The subtypes differ in the hypervariable region of the receptor binding domain of the viral envelope gene. KoRV exists in both endogenous and exogenous forms, with KoRV-A being the only endogenous subtype that is transmitted vertically by integrating into the host’s germline and is inherited in subsequent generations. KoRV-A to M are exogenous subtypes that are transmitted horizontally through somatic cell infection.^3,6,16^ KoRV is ubiquitous in the northern states of Queensland and New South Wales, with a 100% prevalence, but occurs at much lower rates (14.8%–72.2%) in southern populations in Australia depending on the population examined.^36,39^ Some studies have reported lower KoRV viral and proviral loads in southern populations as well as an absence of KoRV infection in certain southern island populations. This suggests KoRV is actively undergoing germline endogenization in the koala genome from the north, despite evidence of limited gene flow between these populations.^7,18,19,23,36,37,39,43^ In 1 study examining northern captive koala populations (*n* = 64), KoRV-A was found in 100% of the koalas analyzed, while the next 2 most prevalent subtypes, KoRV-B and KoRV-D, were found at prevalences of 35.8% and 82.8%, respectively.^20^ Retroviral infections in multiple species have been associated with neoplasms, and thus, the ability of exogenous KoRV subtypes to transduce oncogenes and increase cancer risk is of high interest, particularly because koalas suffer from a relatively high cancer prevalence (5.4%–13.4%).^7,12,24,27^ Lymphoma and leukemia are the most common types of neoplasia reported in both northern and southern populations of koalas, with a region-dependent prevalence of up to 7.5%.^7^ In contrast, lymphoma prevalence can be up to 40% in captive koalas in Australia and internationally.^10,47^

Despite the strong associations of retroviruses with cancer in other species, the complete disease spectrum of KoRV is currently unknown, as there are no studies that definitively prove disease causation by KoRV. However, there is an accumulating body of evidence supporting its role in immunosuppression and neoplasia, particularly as koalas with round cell tumors are documented to have high KoRV proviral loads.^5,7,33,36,44,46^

Osteochondromas are the second most common neoplasia in koalas after lymphoid tumors, with a prevalence of 4.5% in Queensland.^7^ These tumors can undergo malignant transformation and often cause facial distortion, organ compression, pain, and mortality.^4,28,40^ Koalas with osteochondromas have higher KoRV proviral loads, but KoRV’s role in bone neoplasia remains unclear.^7^ While research has focused on KoRV’s link to lymphoid neoplasia and chlamydiosis, further study is needed to understand its oncogenic potential, given welfare concerns and the species’ endangered status.

This study aimed to examine the link between KoRV and bone neoplasia in koalas by quantifying proviral DNA in neoplastic and control bone using molecular techniques and by assessing KoRV replication using serological methods. We hypothesized that neoplastic bone tissue would contain a higher proportion of cells with KoRV protein expression and proviral load compared to healthy bone tissue, thereby providing further insights into the oncogenic role of exogenous KoRV subtypes in this threatened species.

## Materials and Methods

### Sample Collection

Koalas used in this study were opportunistically collected from individuals admitted for autopsy following euthanasia due to terminal chronic disease or trauma or found dead in the wild. Normal femoral and humeral bone tissues were collected (control group) during post-mortem examinations. Samples were also retrospectively retrieved from the archival formalin-fixed, paraffin-embedded (FFPE) bone tumor tissue blocks of the Veterinary Laboratory Services, School of Veterinary Science at the University of Queensland, Gatton Campus. Ethics approval for this study was granted by The University of Queensland Animal Ethics Committee, permit number ANRFA/2023/AE000153 and the Queensland Government Research Permit WA0051629.

### Koala Retrovirus Proviral DNA Real-Time Quantitative Polymerase Chain Reaction

Real-time quantitative polymerase chain reaction (PCR) was used to detect and quantify KoRV proviral DNA in FFPE koala lymphoid tissue, fresh control bone samples, FFPE bone tumor samples, and fresh bone tumor samples. FFPE tissues were deparaffinized using a standard protocol with serial xylene and ethanol washes.^32^ Genomic DNA was extracted from 10 mg lymphoid and ≤25 mg bone tissue samples using the DNeasy Blood and Tissue Kit (Qiagen, catalog No. 69504), following the manufacturer’s instructions. The concentration (ng/µL) was measured using 2 µL of DNA extract for each tissue sample using the NanoDrop 1000 Spectrophotometer (Thermo Fisher Scientific, Waltham, Massachusetts). The PCR primer sets used are listed in Table 1. *β-actin* gene, KoRV *pol* gene, and KoRV subtype-specific *env* gene PCRs were performed. Primers specific to the *β-actin* gene sequence were used as an internal PCR control gene, as it is considered a housekeeping gene that is constitutively expressed in all tissue types and commonly used to normalize gene quantification. This allowed the normalization of KoRV proviral load across different samples. Real-time quantitative PCR was conducted in a CFX96 Touch Real-Time PCR Detection System (Bio-Rad, Hercules, California). PCR reactions were performed in triplicate in 96-well plates with a no template negative control to detect contamination. Reaction mixtures consisted of 2 times master mix (10 µL; PowerUp SYBR Green Master Mix, Applied Biosystems, Waltham, Massachusetts; catalog No. A25742), 800 nM each primer, 3.4 µL water, and 5 µL DNA extract diluted 1/10. PCR cycling conditions were 50°C for 2 minutes, 95°C for 2 minutes, followed by 50 cycles of 95°C for 30 seconds, 59°C for 15 seconds, and 60°C for 1 minute. A melt curve analysis was performed from 60°C to 95°C in increments of 0.5°C.

**Table 1.** Gene targets and primer sequences used for real-time quantitative PCR analysis for koala retrovirus detection.

Gene Target	Primer Sequence	Product Size	Annealing Temperature	Reference
Koala *β-actin*	Fwd 5’-GGAATCCTGTGGAATCCATG-3’	137 bp	60°C	Sarker et al^37^
	Rev 5’-TCTGCATCCTGTCAGCAATG-3’			
KoRV *pol*	Fwd 5’-TTGGAGGAGGAATACCGATTACAC-3’	111 bp	60°C	Szilasi et al^41^
	Rev 5’-GCCAGTCCCATACCTGCCTT-3’			
KoRV-A *env*	Fwd 5’-TCCTGGGAACTGGAAAAGAC-3’	320 bp	59°C	Tarlinton et al^44^
	Rev 5’-GGGTTCCCCAAGTGATCTG -3’			
KoRV-B *env*	Fwd 5’-TCCTGGGAACTGGAAAAGAC-3’	271 bp	59°C	Tarlinton et al^44^
	Rev 5’- GGCGCAGACTGTTGAGATTC -3’			
KoRV-D *env*	Fwd 5’-TCCTGGGAACTGGAAAAGAC-3’	298 bp	59°C	Peauroi et al^31^
	Rev 5’-GRTTCCCCAAGGKCGR-3’			

Abbreviations: Fwd, forward; Rev, reverse; bp, base pairs.

The threshold cycle (C_T_) is the cycle number at which the amplified DNA product has reached a threshold (100 RFU). The mean C_T_ value was calculated for each sample using the triplicate values. The relative gene expression was analyzed using the 2^−ΔΔC^_T_ method as previously described with *β-actin* as the internal control gene.^25^

### Histology

Control bone and neoplastic bone samples were collected from koalas during post-mortem examination and placed in 10% neutral-buffered formalin for tissue fixation for at least 48 hours. Bone tissue samples were decalcified in 15% EDTA solution and 10% formic acid that were changed 2 to 3 times per week until tissues were suitable for trimming. Trimmed tissues were placed in cassettes, stored in 70% ethanol, and underwent overnight processing using an automated tissue processor (Tissue Tek VIP 6 AI-E2; Sakura, the Netherlands). Tissues were embedded in paraffin blocks following standard histological protocols. Tissue blocks were sectioned at 4 µm and mounted onto glass slides for hematoxylin and eosin staining and immunohistochemical processing.

### Immunohistochemistry

Archival FFPE lymphoid tissue was utilized from an adult koala for immunohistochemistry (IHC) optimization, as lymphocytes and lymphoid tissue have been suggested as key sites for KoRV replication.^7^ Immunohistochemical optimization was performed using an automated immunolabeling process with the LINK48 Autostainer (Dako, Denmark). The lymphoid tissue sections were deparaffinized and rehydrated using a series of ethanol and xylene washes with the ST5020 Autostainer (Leica, Australia). Two buffer solutions, a Tris/EDTA buffer at pH 9.0 and a citrate buffer at pH 6.0, were used for heat-induced antigen retrieval with the PT Link Tank (PT200; Dako) at 95°C for 20 minutes. After cooling down for 10 minutes, tissue sections were incubated for 1 hour at room temperature with the primary antibody (polyclonal antibody raised in rabbits against purified recombinant KoRV p30 gag protein expressed in *E. coli*). The antibody was diluted with EnVisionwq FLEX Antibody Diluent (Dako). Serial dilutions of 1:200, 1:400, 1:800, 1:1200, 1:1600, and 1:1800 were used for primary antibody optimization. After endogenous peroxidase inhibition with FLEX peroxidase blocking reagent for 5 minutes, the tissue sections were incubated with Dako horseradish peroxidase goat anti-rabbit, anti-mouse secondary antibody (EnVision FLEX/HRP) for 20 minutes. 3,3’-diaminobenzidine (DAB) and magenta chromogens were both tested in separate optimization runs to determine the ideal chromogen that produced the greatest contrast between antigen immunolabeling and nonspecific background. DAB was incubated on the tissue sections for 10 minutes (EnVision FLEX DAB+ Chromogen). Subsequently, the slides were counterstained with Mayer’s hematoxylin (EnVision FLEX Hematoxylin Dako-Agilent) for 5 minutes as the final step. Tris-buffered saline solution containing Tween 20 was used as a wash buffer (EnVision FLEX Wash Buffer) in between each step. The samples were dehydrated with xylene and ethanol washes and cover slipped. All immunolabeling procedures used a negative control where no primary antibody was incubated on the slide.

Several methods were tested to reduce bone tissue detachment from the slides, following the heat-induced antigen retrieval step of the IHC assay. These included a no antigen retrieval step and enzymatic-induced antigen retrieval with proteinase K (10 µg/ml for 5 minutes at room temperature) and citrate buffer at 95°C for 10 minutes. Reducing the time used for the heat-induced antigen retrieval step resulted in the least amount of tissue detachment and the greatest detection of positive immunolabeling. The optimized IHC assay conditions comprised of 1:1600 primary antibody dilution with low pH citrate antigen retrieval at 95°C for 10 minutes, and the DAB chromogen was applied to all control and neoplastic bone tissues. The negative and positive controls accompanied each IHC run. Photomicrographs obtained during the validation procedure for the selected dilution can be found in Supplemental Fig. S1.

### Immunohistochemistry Assessment

Slides were evaluated by conventional light microscopy, and a semi-quantitative scoring method was used by determining the H-score for each sample.^9^ Images were assessed at 40× magnification (equivalent to an area of 2.37 mm^2^) in 10 fields per section, and the percentage of cells with positive immunolabeling was recorded in each field. Positive immunolabeling was defined as punctate cytoplasmic labeling. A qualitative assessment of the labeling intensity was classified into no labeling (0), weak (1), moderate (2), and strong (3). The percentage of positive cells and the intensity of labeling were combined using the H-score and were calculated using the following formula:

$$\begin{array}{l} H-\mathrm{score}=\left[\left(\%\;\mathrm{at}\;0\right)\times 0\right]+\left[\left(\%\;\mathrm{at}\;1\right)\times 1\right]\\ \;\;+\left[\left(\%\;\mathrm{at}\;2\right)\times 2\right]+\left[\left(\%\;\mathrm{at}\;3\right)\times 3\right]. \end{array}$$

The analysis was performed by a board-certified veterinary pathologist.

### Statistical Analysis

The mean comparative CT (2^−ΔΔC^_T_) was calculated for each tissue sample in the PCR analysis. The Mann-Whitney U test was used to assess significant differences between groups. All statistical analyses were undertaken using STATA. A *P*-value ≤ .05 was considered a statistically significant result.

The data analyzed in this study are available as Supplemental Materials.

## Results

### Diagnoses and Histopathologic Findings

Eleven adult koalas included in this study were submitted from the Moggill Koala Rehabilitation Centre in southeast Queensland and used as the control bone samples (Table 2). There were 5 females and 6 males, with an estimated age ranging from 3.5 to 7 years based on published koala tooth wear charts.^45^ Post-mortem examination and histological findings revealed that 64% (7/11) of koalas that were otherwise healthy died from traumatic injury and 36% (4/11) died from infectious and non-infectious terminal illnesses. Koalas without signs consistent with trauma were suspected of dying due to terminal illnesses or unknown causes, including a hematopoietic tumor, emaciation with sarcoptic mange infestation, and systemic circulatory leukocytosis of unknown etiology. Control koala 11 was under rehabilitative care and euthanized due to terminal illness with chlamydiosis.

**Table 2.** Details of control koalas used in the study (*n* = 11) including identification number, age, sex, and cause of mortality.

ID Number	Age (years)	Sex	Diagnosis
Control koala 1	5.5	F	Blunt force trauma
Control koala 2	4.5	M	Sarcoptic mange and emaciation
Control koala 3	6.5	M	Suspected hematopoietic tumor
Control koala 4	7	F	Blunt force trauma
Control koala 5	3.5	F	Blunt force trauma with underlying ocular and urogenital chlamydiosis
Control koala 6	3.5	M	Blunt force trauma
Control koala 7	4.5	M	Blunt force trauma
Control koala 8	5	M	Blunt force trauma
Control koala 9	5	M	Blunt force trauma
Control koala 10	5.5	F	Systemic circulatory leukocytosis, unknown etiology
Control koala 11	5	F	Ocular and urogenital chlamydiosis

Abbreviations: F, female; M, male.

**Figure 1.**
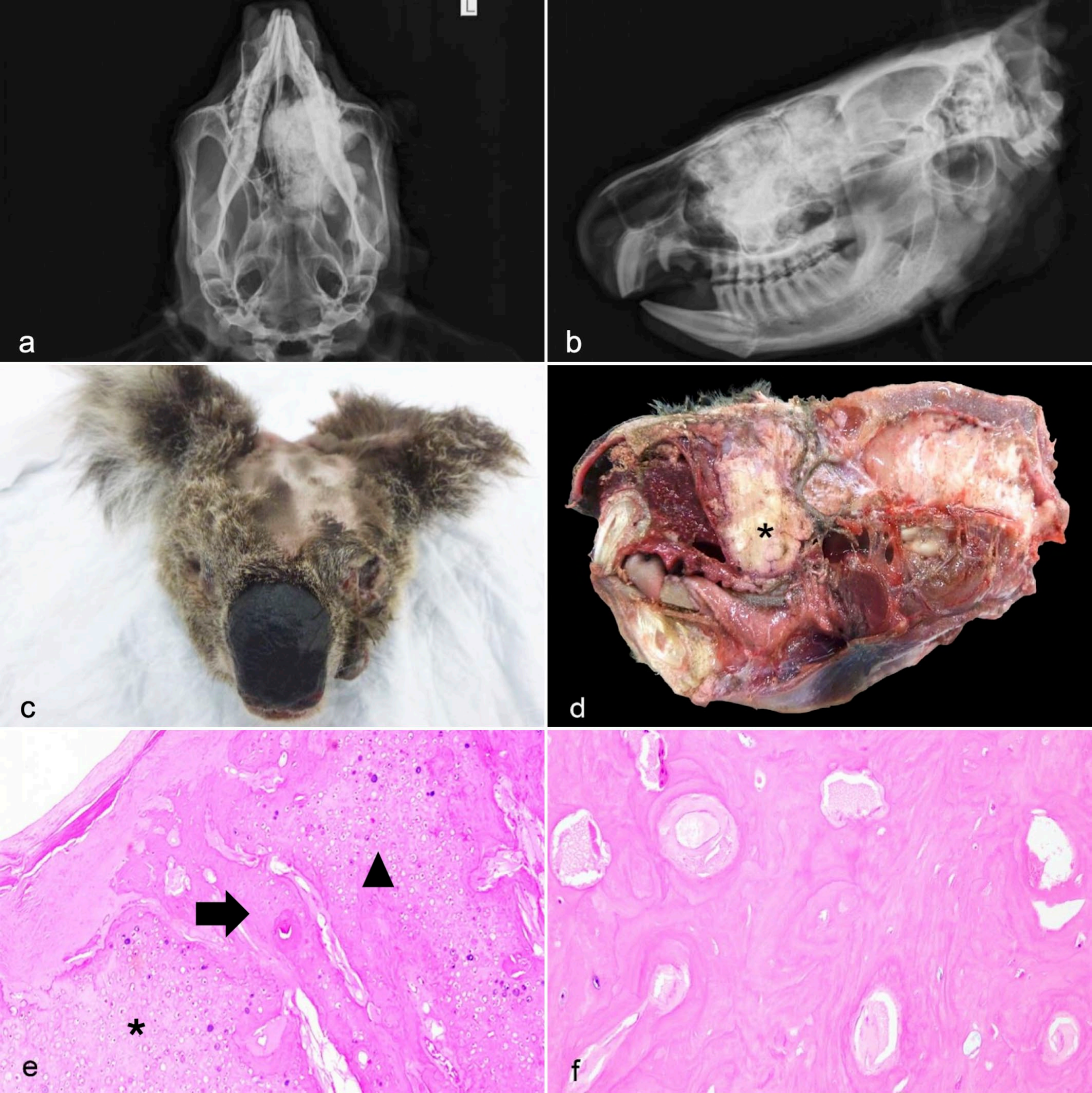
Pathology of intracranial monostotic osteochondroma in sample koala 13. (a) Ventrodorsal radiograph. A well-defined, irregularly shaped expansile but non-infiltrative mass of mixed radio-opaque and lucent densities occupies most of the left frontal sinus and extends into the nasal sinus. (b) Right lateral view radiograph. The mass compresses and displaces the hard palate ventrally. (c) Gross examination, proptosis of the left eye with periocular secretion. (d) Sagittal section of the cranium in which a light tan, hard, well-demarcated mass (asterisk) obliterates the nasal cavity and extends to the rostral braincase and the hard palate. (e) The neoplasm has evidence of osteochondral ossification; its leading edge is formed by a discontinuous cap of cartilaginous tissue (asterisk) and underlying cancellous bone (arrow), as it occurs in the mature form of the tumor. Neoplastic chondrocytes commonly form cartilage tongues (arrowhead) that branch out of the cartilage cap and penetrate the tumor core (not displayed in the photomicrograph). Hematoxylin and eosin (HE). (f) The core of the mass is formed by well-differentiated anastomosing trabeculae of mature lamellar bone. HE. Panels (a) and (b) photo credits: Dr Julien Grosmaire, 2023.

**Figure 2.**
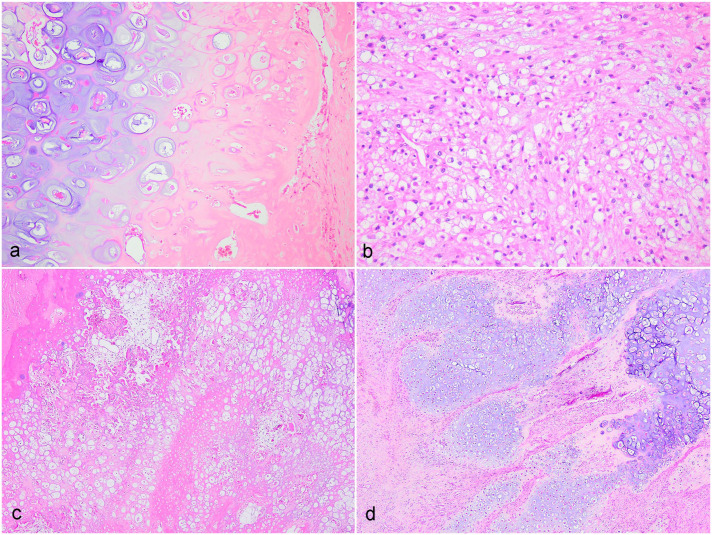
Histopathological features of osteosarcomas and chondrosarcomas in koalas. Hematoxylin and eosin. (a) Osteosarcoma, right shoulder, sample koala 7. Neoplastic osteoid matrix with a mixture of cartilage and collagen. (b) Osteosarcoma, left iliac crest, sample koala 14. Neoplastic, pleomorphic osteoblasts are observed effacing the iliac crest. (c) Chondrosarcoma, temporal bone, sample koala 2. Sheets of neoplastic chondrocytes are embedded within a chondroid matrix forming a disorganized, infiltrative mass. (d) Chondrosarcoma, left scapula/spine, sample koala 12. Neoplastic chondrocytes are observed either grouped in chondrones or in large islands comprised of disorganized hyaline cartilage irregularly infiltrating adjacent tissue.

Twelve archival FFPE bone neoplasia samples were collected between 2001 and 2022 (Table 3), including osteochondromas displaying features of early and mature stages (Fig. 1),^28^ osteosarcomas (Fig. 2a, b), and chondrosarcomas (Fig. 2c, d). The 13th (Fig. 1) and 14th bone tumor samples were collected from koalas found deceased in the wild. Eleven of the 14 tumors (79%) were located in the skull. An estimated age was not recorded for most individuals; however, all were adult koalas.

**Table 3.** Details of koalas with bone tumors used in the study (*n* = 14) including identification number, age, sex, bone tumor classification, and location of tumor.

ID Number	Age (years)	Sex	Diagnosis	Tumor Location
Sample koala 1	5	M	Chondrosarcoma	Mandible
Sample koala 2	Unknown	Unknown	Chondrosarcoma	Temporal bone
Sample koala 3	Unknown	Unknown	Osteochondroma	Mandible
Sample koala 4	Unknown	M	Osteochondroma	Oronasal and hard palate
Sample koala 5	Unknown	M	Osteochondroma	Frontal and parietal bones
Sample koala 6	Unknown	F	Osteochondroma	Right side of nose
Sample koala 7	Unknown	Unknown	Osteosarcoma	Right shoulder
Sample koala 8	Unknown	Unknown	Osteosarcoma	Mandible
Sample koala 9	Unknown	Unknown	Chondrosarcoma	Hard palate
Sample koala 10	Unknown	Unknown	Osteochondroma	Intranasal septum
Sample koala 11	Unknown	Unknown	Osteochondroma	Nasal cavity
Sample koala 12	Unknown	F	Chondrosarcoma	Left scapula and spine
Sample koala 13	5 yr	F	Osteochondroma	Left cranium
Sample koala 14	Unknown	M	Osteosarcoma	Left iliac crest

Abbreviations: M, male; F, female.

### Koala Retrovirus Proviral DNA Analyses Using Real-Time Quantitative Polymerase Chain Reaction

To detect and quantify KoRV proviral DNA in bone samples, DNA was extracted, and real-time quantitative PCR analyses were performed for 4 gene targets: KoRV *pol*, KoRV-A *env*, KoRV-B *env*, and KoRV-D *env*. KoRV *pol* proviral DNA and *β-actin* were detected in 25/25 control and bone tumor tissue samples. The universal KoRV *pol* primer set in the PCR analysis allowed for the detection of all KoRV subtypes, as the *pol* gene is conserved between all subtypes. As expected, KoRV proviral DNA was detected in all samples analyzed as all Queensland koalas are known to harbor the endogenous KoRV-A subtype.^36,39^

To compare the KoRV proviral DNA quantities between control bones and bone tumors, the mean relative KoRV proviral load was calculated using the 2^−ΔΔC^_T_ method (Table 4). The control bone samples had a median relative *pol* gene proviral load of 1.00 (interquartile range [IQR] = 0.93–1.15), and the bone tumor samples had a median relative proviral load of 1.05 (IQR = 0.91–1.26). The median relative proviral load of KoRV DNA was not significantly different between the control bones and bone tumor samples (*P* = .687, Mann-Whitney U test; *n* = 11 controls, *n* = 14 bone tumors).

**Table 4.** Relative proviral load data analysis using the 2^−ΔΔC^_T_ method showing the relative fold change in the KoRV *pol* gene and subtype-specific KoRV *env* genes normalized to the internal control gene, *β-actin*, and relative to the mean ΔC_T_ of the control bone samples.

Gene	Tissue Type	Median Relative Proviral Load (Interquartile Range)	*P-*value^a^
Universal KoRV *pol*	Control bone	1.00 (0.93, 1.15)	.687
	Bone tumor	1.05 (0.91, 1.26)	
KoRV-A *env*	Control bone	1.13 (0.80, 1.60)	.**025**
	Bone tumor	0.73 (0.65, 0.97)	
KoRV-B *env*	Control bone	3.38 (0.33, 76.40)	.501
	Bone tumor	31.14 (0.16, 69.57)	
KoRV-D *env*	Control bone	1.52 (0.88, 3.00)	.767
	Bone tumor	1.47 (0.32, 2.75)	

a Bold values are considered significant (Mann-Whitney U test, *P* < .05).

**Figure 3.**
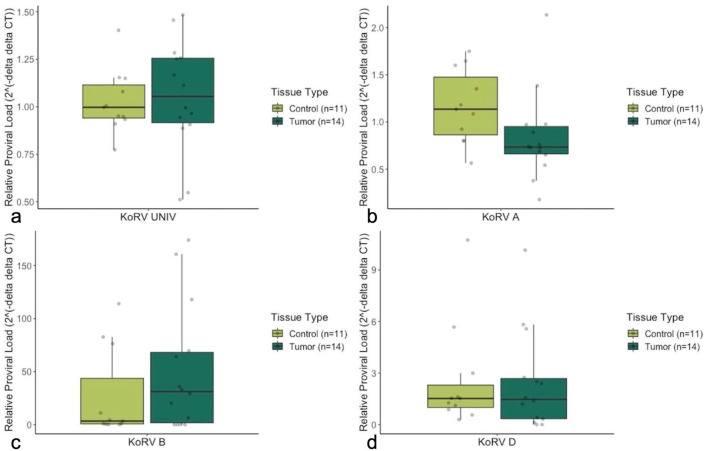
Box and whisker plots of relative koala retrovirus (KoRV) proviral loads between healthy control bones (*n* = 11) and bone tumor samples (*n* = 14) measured by real-time quantitative PCR analysis. (a) Universal KoRV *pol* gene, (b) KoRV-A specific *env* gene, (c) KoRV-B specific *env* gene, and (d) KoRV-D specific *env* gene. Values are normalized to koala *β-actin* and expressed as relative fold change compared to the mean value of healthy controls. Proviral *env* gene load of KoRV-A is significantly higher (*P* = .025) in control bones compared to bone tumor samples. The median proviral load of KoRV-B is higher in bone tumor samples compared to the control bone group; however, the difference is statistically insignificant (*P* = .501). The median proviral loads of KoRV *pol* and KoRV-D show no significant difference between the 2 groups.

To determine whether certain KoRV subtypes had a higher proviral load in bone tumors compared to control bones, subtype-specific primers were used to measure proviral loads (Fig. 3). PCR primers designed to amplify part of the hypervariable region of the KoRV *env* gene were used to differentiate between 3 KoRV subtypes: KoRV-A, KoRV-B, and KoRV-D. These 3 KoRV subtypes have been described as the most prevalent subtypes detected in captive koala populations.^21^ KoRV-A *env* gene was detected in 25/25 (100%) koalas tested. Control bones had a significantly higher KoRV-A relative *env* gene proviral load compared to bone tumors (*P* = .025). KoRV-B *env* was detected in 11/11 (100%) control koalas and 11/14 (79%) koalas with bone tumors. The bone tumor group had a higher median relative KoRV-B *env* gene proviral load compared to the control group, but the difference was not significant (*P* = .501). KoRV-D *env* gene was detected in 11/11 (100%) control koalas and 12/14 (86%) koalas with bone tumors. There was no significant difference in KoRV-D relative *env* gene proviral load between control bones and bone tumors (*P* = .767). The relative proviral load for all gene targets in each sample is presented in Supplemental Table S1.

A melt curve analysis confirmed the presence of KoRV proviral DNA and *β-actin* gene products in FFPE koala lymphoid tissue, which was subsequently used as a positive control for IHC analysis. The C_T_ values and melting temperatures for each sample are presented in Supplemental Table S2.

### Immunohistochemistry Assay Development for the Detection of Koala Retrovirus in Bone Tissue

**Figure 4.**
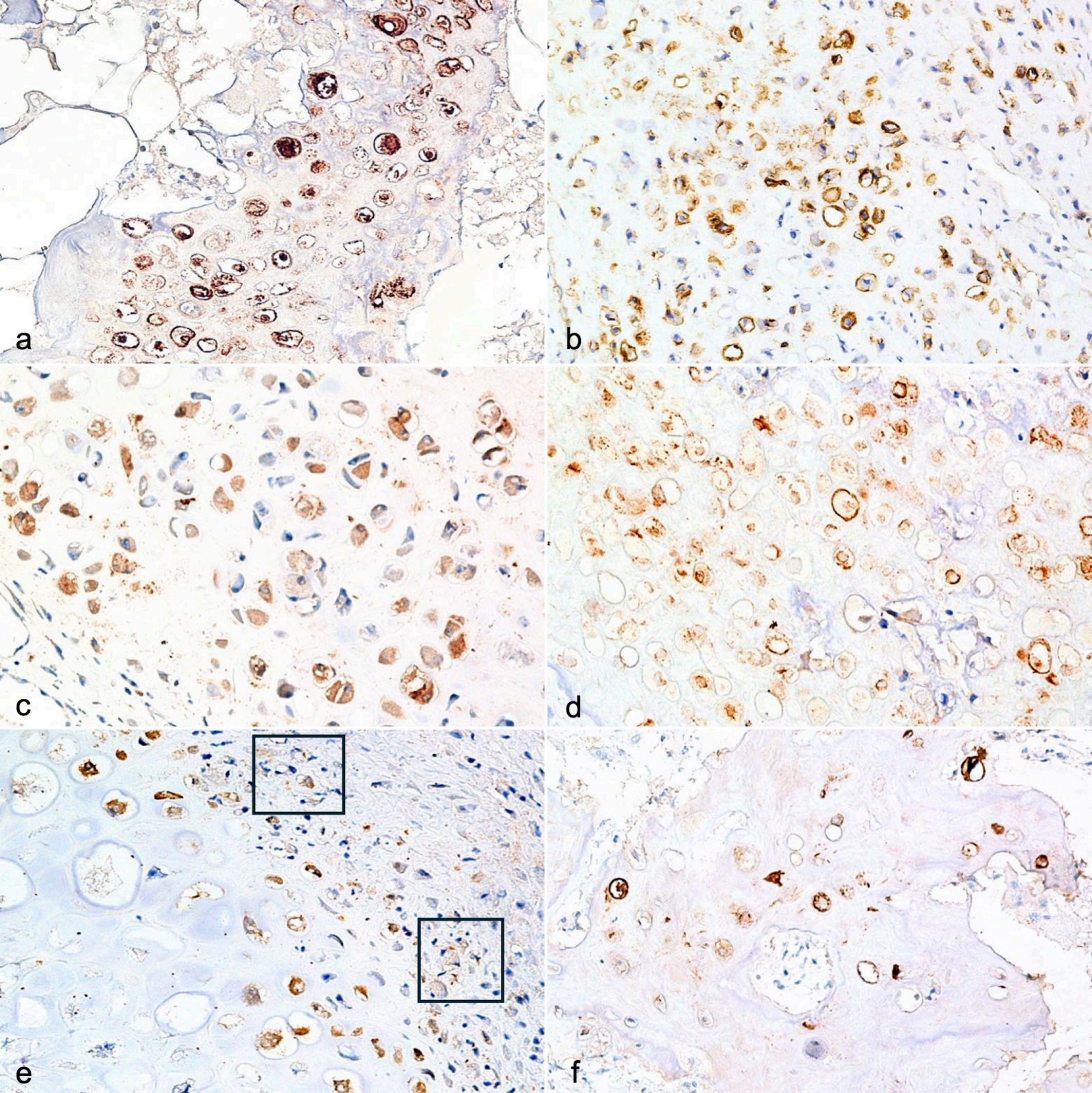
Immunohistochemical detection of koala retrovirus (KoRV) capsid protein expression in bone neoplasms. Cytoplasmic and nuclear immunolabeling can be seen in the neoplastic chondrocytes within lacunae in chondrosarcomas [(a)–(c), sample koalas 2, 9, and 12, respectively], in osteochondromas [(d) and (e), sample koalas 11 and 4, respectively], and within neoplastic osteoblasts in an osteosarcoma [(f), sample koala 7]. Immunolabeling is also observed in perilesional mesenchymal cells ((e), black squares). Over half of neoplastic cells have strong labeling in chondrosarcomas and osteosarcomas; this contrasts with the decreased proportion and reduced immunolabeling intensity of neoplastic cells in osteochondromas. Anti-KoRV gag immunohistochemistry.

To determine the presence of KoRV viral protein in bone samples, an optimized IHC analysis was performed. KoRV gag capsid protein immunolabeling was evaluated in bone tumors and control bone samples with 25 total samples analyzed. IHC for the KoRV gag protein detected positive cytoplasmic and nuclear immunolabeling of tumor cells in all bone tumor samples (14/14, 100%). Positively labeled cell types included chondrocytes, osteocytes, and osteoblasts (Fig. 4). The proportion of immunolabeled cells in each field ranged from 0% to 100%, with weak to strong labeling intensities (Supplemental Table S3). Due to tissue fragmentation, the 10 fields were not evaluated consecutively, and assessments were made in regions where the tumor tissue encompassed the entire field. When multiple slides were present for the same bone tumor sample, the slide with the least amount of tissue fragmentation was selected for assessment. There was 1 sample where the degree of bone fragmentation caused by the IHC assay required analysis of the bone remnant remaining, which included osteocytes. The overall mean percentage of positive immunolabeling in the bone tumor samples was 50.4 ± 23.2% (mean ± standard deviation). The highest mean percentage of tumor cell labeling was observed in osteosarcomas (*n* = 3, 73.0 ± 29.8%), followed by chondrosarcomas (*n* = 5, 51.6 ± 13.7%) and osteochondromas (*n* = 6, 38.0 ± 20.1%). The median H-score in control samples was 10 (IQR = 5–40), and in bone tumor samples, it was 108.5 (IQR = 78–156). The tumor group had a significantly higher H-score when compared to the control group (*P* < .001). When comparing malignant chondrosarcomas and osteosarcomas (median = 139.5 [IQR = 90–190.5]) to benign osteochondromas (median = 71 [IQR = 18–124]), the H-score was marginally higher in malignant tumors (*P* = .08).

## Discussion

Captive and wild koala populations are significantly impacted by a high cancer prevalence, and KoRV has previously been shown to be associated with increased cancer rates, particularly lymphoma and leukemia.^5,7,33,36,44,47^ In this study, we provide insights into the oncogenic potential of KoRV to induce bone tumors.

Previous studies have demonstrated that koalas with neoplasia have higher plasma KoRV viral RNA loads compared to healthy koalas as well as increased KoRV proviral load in the peripheral blood of koalas with osteochondromas.^7,44^ Proviral KoRV loads have also been observed to increase coinciding with a leukemia diagnosis in a captive koala (personal communication, K. Chappell). However, no literature detecting the presence of KoRV-infected cells in bone tumors has been reported thus far. To expand on these studies, we examined KoRV proviral load in healthy bone tissues from 11 koalas and bone tumor tissues from 14 koalas using real-time quantitative PCR, allowing us to compare KoRV subtype-specific proviral loads between the 2 groups. KoRV proviral DNA was detected using the universal *pol* gene in all samples analyzed in the study, confirming the presence of reverse-transcribed retroviral DNA, the first step of the retroviral replication process. All Queensland koalas harbor the KoRV provirus in their genome, as KoRV-A is endogenously transmitted in the germline.^36,39^ When examining all KoRV subtypes collectively (*pol* gene), as well as KoRV-B and KoRV-D specifically (*env* gene), we found no significant difference in proviral load between bone tumors and healthy bone. Interestingly, control bones had a significantly higher KoRV-A *env* gene proviral load than tumor samples, suggesting a possible role of endogenously inherited KoRV-A in the pathogenesis of bone tumor development, providing a host defense mechanism and inhibiting tumor development. As described in a previous study, KoRV was found to recombine with an endogenous retroelement called *Phascolarctos* endogenous retrovirus. The resultant degraded recombinant KoRV lacks most of the retroviral genes, thereby preventing it from producing functional retroviral proteins needed for viral replication.^26^ All Queensland koalas in that study (48/48) tested positive for KoRV and recombinant KoRV. We observed a higher median KoRV-B proviral load in bone tumor samples compared to controls (although not significant at *P* < .05), aligning with previous studies linking KoRV-B to neoplasia. However, despite the observed trend, our result was not statistically significant, possibly reflecting the small sample size used in this study. Similarly, the median KoRV-D proviral load in control and bone tumor samples showed no significant difference. Quantification of proviral DNA between tumoral and non-tumoral tissue is a complex task, as many factors play into viral oncogenesis, including but not limited to, efficiency of viral integration, level of viral replication, and the mechanisms used by the virus to drive oncogenesis.

Although KoRV provirus detection in both groups was expected, we did not anticipate to find no significant relationship between proviral load and bone tumor tissue based on previous findings. A possible explanation for this finding could be that the provirus detected in healthy bone is not transcribed into viral RNA, and therefore, the virus is not actively replicating in cells. Similar to feline leukemia virus (FeLV)-infected cats, where proviral DNA integrates into the genome but does not always produce viral RNA, KoRV may exist in a latent state in healthy bone.^15^ Previous research supports this hypothesis, demonstrating that while fragments of KoRV proviral DNA were present in 99% of South Australian koalas, only 51.2% of the same koalas had detectable viral RNA.^36^ Another study found that South Australian koalas that tested positive for proviral DNA were replication defective, such that portions of the provirus were not being transcribed into viral RNA.^42^ Further studies using reverse-transcription PCR or RNA in situ hybridization could clarify whether KoRV is actively replicating in bone tumor cells.

To our knowledge, this is the first report of KoRV immunohistochemical detection in koala bone tumors. An IHC assay was optimized using KoRV provirus-positive koala lymphoid tissue and detected KoRV capsid protein in neoplastic bone cells. A positive control needed to be established for the KoRV gag primary antibody to confirm that positive immunolabeling was specific to the KoRV viral capsid protein rather than producing nonspecific false positive labeling. This was accomplished by using real-time quantitative PCR to detect the presence of KoRV provirus in koala lymphoid tissue that was subsequently used as a positive control tissue. After applying the validated KoRV gag IHC assay to bone tumor samples, KoRV viral capsid protein was observed in the cytoplasm of osteoblasts, chondroblasts, and osteocytes, as we hypothesized. Unexpectedly, we also detected immunolabeling in the surrounding osteoid and chondroid matrix, a phenomenon previously reported in FeLV-infected cats.^1^ This could be a result of positive labeling in the cytoplasmic projections of chondrocytes that extend into the adjacent matrix. Another possible explanation is the deposit of KoRV capsid protein during the production of chondroid or osteoid matrix by infected tumor cells. Our study demonstrated that bone tumor samples exhibited a significantly higher H-score compared to healthy bone tissues. This is an important finding, as it suggests that KoRV is actively replicating in bone tumor cells at a significantly higher degree than in healthy bone cells.

Our results demonstrated that osteosarcomas and chondrosarcomas had a higher percentage of immunolabeled tumor cells compared to osteochondromas. This could be explained by the malignant nature of osteosarcomas and chondrosarcomas having a higher cell replication potential. Whether the greater number of cells with retroviral protein expression is a predisposing factor of malignant transformation or malignant transformation of the tumor led to increased expression of retroviral protein is undetermined and beyond the scope of the current study. Additionally, there were samples of bone tumors where immunolabeling concentrated in the perilesional mesenchymal cells, which could correspond to neoplastic periosteal or perichondrium-like connective tissue (Fig. 4e). Similarly, a previous study reported undifferentiated sarcomatous cells at the margins of an osteosarcoma in a koala, suggesting malignant transformation of a mostly benign mass occurring at the periphery of the tumor.^40^ The increased immunolabeling observed in these cells may be due to retroviral replication concentrating in cells where the tumor is expanding from and undergoing malignant transformation.^28^

This study was limited by a small sample size due to the opportunistic nature of sample collection in a short timeframe. Bone tissue from the femur and humerus was collected as the control samples. Samples from long bones were collected, as osteochondromas most commonly affect the bones of the skull, long bones, clavicle, ribs, and pelvis.^14^ It is presumed in our study that KoRV capsid protein expression is consistent across all healthy skeletal tissues in koalas. Future studies can consider assessing control bone tissue from multiple locations of the koala to assess whether differential KoRV protein expression exists depending on the location of the bone.

Besides the ability of exogenous retroviruses to increase cancer risk by oncogene transduction, the role of retroviruses in immunosuppression induction and neoplasia development has been well documented in the literature. FeLV, a retrovirus infecting cats, and human immunodeficiency virus have been shown to cause a decline in the CD4+ lymphocyte cell population, thereby reducing the immune defense system, a known oncogenic risk factor.^8,17^ Gibbon ape leukemia virus, a retrovirus determined to be most phylogenetically related to KoRV, has been reported to induce leukemia in gibbons.^13,22^ Similarly, FeLV infection has been strongly correlated with leukemia and lymphoma in cats.^22,38^ In addition, several studies have suggested an involvement of FeLV in osteochondromatosis in cats, with 1 study using in situ hybridization to visualize the presence of FeLV infection in bone tumor cells.^34,41^ Other examples of retroviruses associated with bone neoplasms include multicentric skeletal sarcomas associated with type C retrovirus in hedgehogs and a case of polyostotic osteosarcoma associated with avian leukosis virus in a bare-faced curassow.^30,31^ Previous studies have demonstrated an association between certain exogenous KoRV subtypes and KoRV viral load with neoplasia.^2,33,47^ Further research into the association of KoRV subtypes with primary bone neoplasia development would be informative to assess whether koalas infected with certain KoRV subtypes should be restricted from breeding programs due to their susceptibility to developing neoplasia and thus increased mortality risk. Several studies have shown that KoRV-B can be exogenously transmitted between dam and joey.^21,33,47^ The primary antibody used in the IHC analysis of our study detected the viral capsid protein transcribed from the conserved *gag* gene, thereby allowing the detection of all KoRV subtypes. Future studies could utilize antibodies that detect KoRV subtype-specific *env* proteins to elucidate whether certain subtypes are more likely to be found in neoplastic cells. Further research is required to understand the subtype expression patterns that may play a role in bone tumor oncogenesis.

This study utilized 2 different analysis platforms to detect KoRV, IHC, and real-time quantitative PCR, providing valuable complementary information about KoRV replication in bone tumor tissue. A major advantage of using IHC compared to PCR is the visualization and localization of viral protein products in infected cells. Using both assays in conjunction allowed us to localize replicating virus in neoplastic tissue and objectively determine the quantity of provirus present in the same tissue sample.

Findings from this study suggest that KoRV may have a role in the pathogenesis of bone tumors in koalas. The newly developed and validated IHC assay provided a step forward in the investigation of the role of KoRV in bone tumor oncogenesis. This assay can be used in future research for further investigation into the visualization and localization of KoRV viral protein and thus, the involvement of KoRV in neoplasms of different cell origins. By gaining a greater understanding of the full disease spectrum of KoRV, these findings may help inform intervention strategies on managing the threat of disease on koala health and population viability, such as guiding translocations and breeding programs.

## Supplemental Material

sj-pdf-1-vet-10.1177_03009858251403173 – Supplemental material for Investigating the association between koala retrovirus and primary bone neoplasia in koalas (Phascolarctos cinereus) using real-time PCR and a novel immunohistochemistry assay Supplemental material, sj-pdf-1-vet-10.1177_03009858251403173 for Investigating the association between koala retrovirus and primary bone neoplasia in koalas (Phascolarctos cinereus) using real-time PCR and a novel immunohistochemistry assay by Carmen Chu, Lee McMichael, Joanne Gordon, Chiara Palmieri, Joanne Meers, Joerg Henning and Viviana Gonzalez-Astudillo in Veterinary Pathology
