# Supplementary material for: Investigating the association between koala retrovirus and primary bone neoplasia in koalas (Phascolarctos cinereus) using real-time PCR and a novel immunohistochemistry assay
Source: Vet Pathol. 2025 Dec 26;63(4):664–75. doi: 10.1177/03009858251403173 (PMC13254151; doi:10.1177/03009858251403173)
Supplement: sj-pdf-1-vet-10.1177_03009858251403173 – Supplemental material for Investigating the association between koala retrovirus and primary bone neoplasia in koalas (Phascolarctos cinereus) using real-time PCR and a novel immunohistochemistry assay [file sj-pdf-1-vet-10.1177_03009858251403173.pdf]

Supplemental Materials

Investigating the association between koala retrovirus and primary bone neoplasia in koalas (*Phascolarctos cinereus)* using real-time PCR and a novel immunohistochemistry assay

Carmen Chu, Lee McMichael, Jo Gordon, Chiara Palmieri, Joanne Meers, Joerg Henning, Viviana Gonzalez-Astudillo

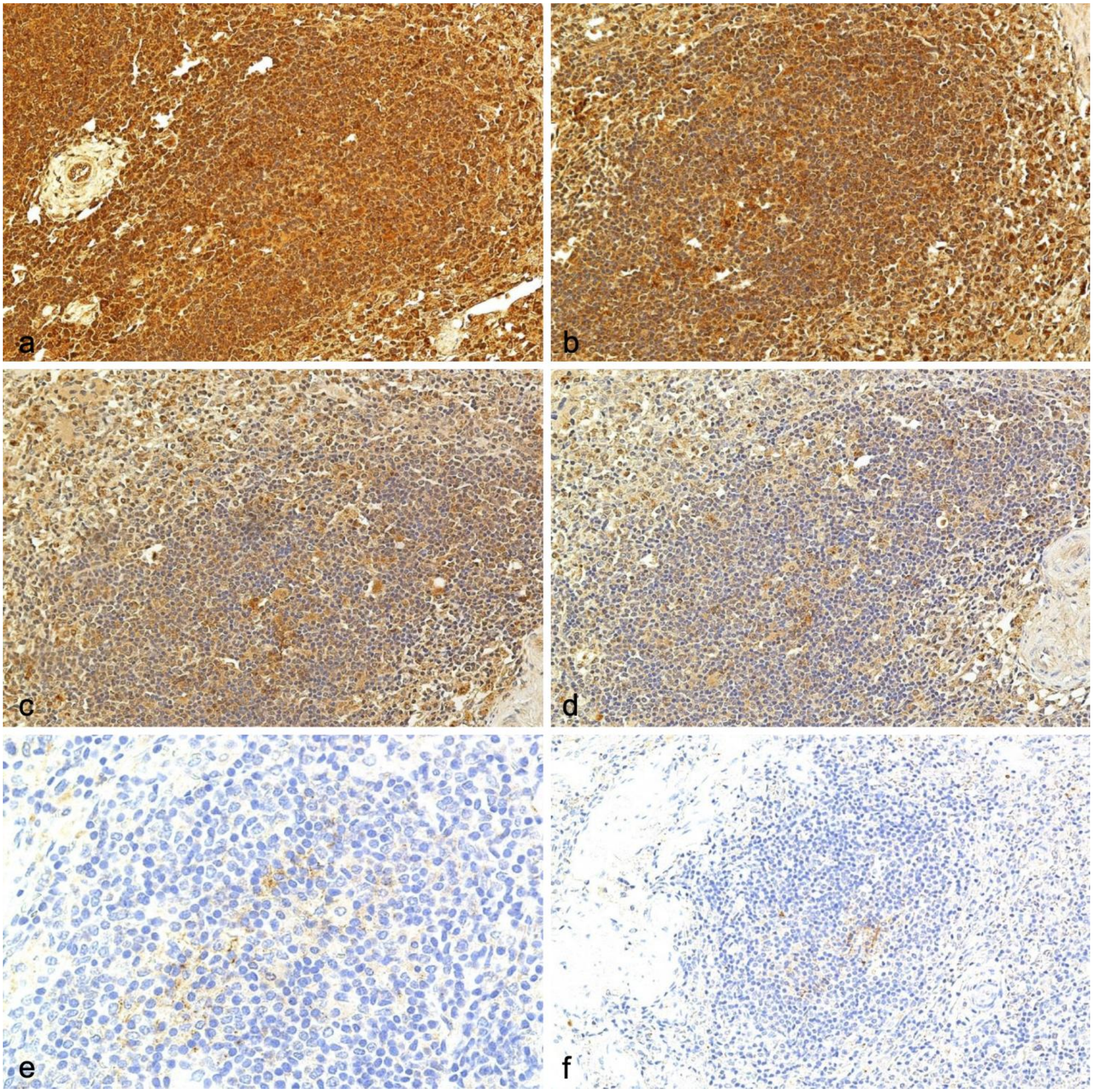

Supplemental Figure S1. Investigation into the immunolabeling of koala retrovirus (KoRV) capsid protein (cytoplasmic) marker in koala lymphoid tissue. Serial dilutions of the primary antibody against the p30 KoRV gag protein were used for the immunohistochemistry optimization procedure including 1:200 (**a**), 1:400 (**b**), 1:800 (**c**), 1:1200 (**d**), 1:1600 (**e**), and 1:1800 (**f**). A primary antibody dilution of 1:1600 revealed optimal immunolabeling with reduced nonspecific background and was subsequently applied to bone tissue samples.

Supplemental Table S1. Relative koala retrovirus (KoRV) proviral load data analysis using the $2^{-\Delta\Delta C_T}$ method showing the relative fold change normalized to the internal control gene, *β-actin*, and relative to the mean ΔC_T of the control bone samples.

ID number	Relative Proviral Load			
	**KoRV *pol***	**KoRV-A**	**KoRV-B**	**KoRV-D**
Control koala 1	0.95	1.18	4.41	0.29
Control koala 2	0.91	1.14	0.12	1.61
Control koala 3	1.15	1.75	76.40	10.74
Control koala 4	1.15	1.65	3.38	0.88
Control koala 5	1.08	0.80	114.08	1.52
Control koala 6	1.40	0.56	11.00	1.26
Control koala 7	0.77	0.92	1.02	1.12
Control koala 8	1.00	1.09	0.15	3.00
Control koala 9	0.93	0.80	0.33	0.56
Control koala 10	0.95	1.35	82.70	5.68
Control koala 11	1.00	1.60	0.97	1.54
Sample koala 1	0.51	0.18	0.04	0.00
Sample koala 2	0.96	1.38	0.00	5.82
Sample koala 3	1.11	0.97	117.99	2.75
Sample koala 4	0.55	0.38	0.01	0.00
Sample koala 5	0.94	0.73	69.57	0.32
Sample koala 6	0.89	0.73	174.04	0.41
Sample koala 7	1.00	0.65	35.91	1.38
Sample koala 8	1.28	0.74	29.31	2.51
Sample koala 9	0.91	0.76	20.23	1.20
Sample koala 10	1.26	0.98	32.97	2.40
Sample koala 11	1.17	0.69	64.22	1.57
Sample koala 12	1.25	0.89	160.74	10.15
Sample koala 13	1.46	2.13	0.16	5.58
Sample koala 14	1.48	0.54	6.35	0.12

Supplemental Table S2. Mean C_T values and melt temperatures (MT) from real-time PCR analysis of control bone and bone tumor samples using koala retrovirus (KoRV) *pol* and subtype specific KoRV *env* primers.

ID number	KoRV *pol* C_T	KoRV *pol* MT (°C)	KoRV-A *env* C_T	KoRV-A *env* MT (°C)	KoRV-B *env* C_T	KoRV-B *env* MT (°C)	KoRV-D *env* C_T	KoRV-D *env* MT (°C)
Control koala 1	18.93	82.50	18.66	87.00	29.46	86.83	29.16	86.67
Control koala 2	18.05	82.50	17.77	87.00	33.67	86.67	25.76	87.00
Control koala 3	15.76	82.50	15.20	87.00	22.45	86.50	21.07	86.83
Control koala 4	15.93	82.50	15.46	87.00	27.11	87.00	24.84	87.00
Control koala 5	16.30	82.50	16.78	87.00	22.32	87.50	24.34	87.00
Control koala 6	17.63	82.50	18.99	87.00	27.40	87.00	26.32	87.00
Control koala 7	16.76	82.50	16.54	87.00	29.09	86.50	24.76	87.00
Control koala 8	16.24	82.50	16.16	87.00	31.74	86.50	23.19	87.00
Control koala 9	17.20	82.50	17.47	87.00	31.45	86.67	26.46	86.50
Control koala 10	16.24	82.50	15.78	87.00	22.54	87.00	22.19	86.83
Control koala 11	16.93	82.50	16.31	87.00	29.72	87.00	24.85	86.00
Sample koala 1	34.54	82.50	36.10	87.00	51.00	n/a	51.00	n/a
Sample koala 2	29.03	82.50	28.55	87.00	51.00	n/a	34.97	86.50
Sample koala 3	29.04	82.50	29.28	86.83	35.05	87.00	36.27	86.25
Sample koala 4	32.79	82.50	33.37	86.83	51.00	n/a	51.00	n/a
Sample koala 5	28.55	82.50	28.96	86.83	35.09	87.00	38.62	87.00
Sample koala 6	29.89	82.50	30.22	87.00	35.02	87.00	39.55	87.50
Sample koala 7	25.23	82.50	25.89	87.00	32.80	86.67	33.30	86.50
Sample koala 8	26.60	82.50	27.45	86.83	34.83	86.50	34.17	86.50
Sample koala 9	27.61	82.50	27.90	87.00	35.87	87.00	35.73	86.67
Sample koala 10	27.03	82.50	27.43	87.00	35.05	86.75	34.63	86.33
Sample koala 11	28.32	82.50	29.12	86.50	35.28	86.50	36.43	86.75
Sample koala 12	30.01	82.50	30.55	86.50	35.75	86.50	35.53	86.50
Sample koala 13	17.48	82.50	16.97	87.00	33.37	86.50	24.08	87.50
Sample koala 14	24.56	82.50	26.06	87.00	35.20	87.00	36.68	87.00

Abbreviation: n/a, not applicable

Supplemental Table S3. Assessment of positive immunolabeling in control bone and bone tumor samples from the immunohistochemistry analysis.

ID number	Mean % immunolabeled cells	Labeling intensity	H-Score
Control koala 1	5	Weak	5
Control koala 2	10	Weak	10
Control koala 3	5	Weak	5
Control koala 4	5	Weak	5
Control koala 5	5	Weak	5
Control koala 6	15	Moderate	30
Control koala 7	20	Moderate	40
Control koala 8	20	Moderate	40
Control koala 9	20	Moderate	40
Control koala 10	15	Moderate	30
Control koala 11	40	Weak	40
Sample koala 1	52	Strong	156
Sample koala 2	41	Moderate	82
Sample koala 3	16	Weak	16
Sample koala 4	32	Moderate	64
Sample koala 5	62	Moderate	124
Sample koala 6	18	Weak	18
Sample koala 7	41	Strong	123
Sample koala 8	78	Moderate	156
Sample koala 9	75	Strong	225
Sample koala 10	47	Moderate	94
Sample koala 11	39	Moderate	78
Sample koala 12	43	Moderate	86
Sample koala 13	61	Strong	183
Sample koala 14	100	Strong	300
